# Comparison of emergency airway management techniques in the performance of emergent Cricothyrotomy

**DOI:** 10.1186/s12245-022-00427-3

**Published:** 2022-05-30

**Authors:** Nicholas George, Gabriel Consunji, Jordan Storkersen, Fanglong Dong, Benjamin Archambeau, Richard Vara, Jan Serrano, Reza Hajjafar, Louis Tran, Michael M. Neeki

**Affiliations:** 1 Department of Emergency Medicine, Arrowhead Regional Medical Center, 400 N. Pepper Ave., Colton, CA 92324 USA; 2 Department of Surgery, Arrowhead Regional Medical Center, 400 N Pepper Ave., Colton, CA 92324 USA; 3 California University of Science and Medicine, 1501 Violet St., Colton, CA 92324 USA

**Keywords:** Cricothyrotomy, Emergent cricothyrotomy, Emergency airway, Cricothyrotomy techniques

## Abstract

**Introduction:**

Emergent cricothyrotomy (EC) is a rare and lifesaving procedure to secure a difficult airway when other methods have failed. Many techniques have been discussed in the literature. This study aimed to identify major techniques used to perform EC in a regional trauma center and evaluate outcomes associated with the techniques.

**Methods:**

Patients who underwent EC at Arrowhead Regional Medical Center between 1-1-2009 and 1-1-2019 were reviewed for eligibility for this study. Patients’ data were extracted from the trauma database. Chi-square tests were conducted to assess the difference on variables between the techniques.

**Results:**

A total of 51 (0.17%) of these patients required EC and were included in the database. The two most prevalent techniques were the scalpel-bougie-tube (SBT) and the surgical cricothyrotomy technique (SCT). More than half (*n* = 27, 52.9%) of the cohort received the SBT. There was no statistically significant difference between the two techniques with regards to demographic variables, including age (*p* = 0.7528), injury severity score (ISS, *p* = 0.896), gender (*p* = 0.3709), and race (*p* = 0.8935). However, the SCT group had a statistically higher Glasgow Coma Scale **(**GCS) than the SBT group (*p* = 0.0036). There was no statistically significant difference in mortality or complications between these two groups (*p* = 0.2172 for mortality).

**Discussion:**

Two techniques of EC were identified as preferred techniques. Both procedures were successful in securing an emergency airway, noting a difference in the time to completion of the two techniques. Given the rarity of the procedure, practitioners may choose the method based on their training and the availability of appropriate instruments.

## Introduction

An emergent cricothyrotomy (EC) is considered the last resort for patients in which attempts for nasopharyngeal or oropharyngeal intubation and ventilation have been unsuccessful or are contraindicated [1]. This procedure may be necessary in patients with significant maxillofacial trauma, oropharyngeal obstruction, or those who cannot be successfully intubated for other reasons [1–3]. Failure to intervene on these critically ill patients may cause profound hypoxia, resulting in permanent disability or death. Patients requiring this intervention are rare and published data are scarce [4]. The incidence of EC has been estimated to be nearly 1–3% during trauma care, 0.4% in non-trauma emergency conditions, and 0.003% in the operating room [5–7]. Stephens and colleagues noted that of 32,000 trauma patients requiring intubation within 24 hours of admission, only 17 had undergone EC, an overall incidence of 0.05% [8].

Emergent cricothyrotomy carries significant risks to patients, including bleeding, infection, and iatrogenic injury to the proximal anatomical structures such as the thyroid arteries, thyroid gland, esophagus, or recurrent laryngeal nerve [1]. Each iatrogenic injury may also cause a long-term complication for the patient that requires further management [1]. Patients with notable morbidity also have increased risk of laryngeal scarring and/or stenosis, which may impair speech and breathing [1]. A meta-analysis of 20 studies encompassing 1628 EC cases found a 3.9% rate of laryngotracheal sequelae requiring further intervention, with a 1.7% rate of chronic subglottic stenosis [9]. Furthermore, as EC is not intended to establish a long-term airway, the patient will require a conversion to a formal tracheostomy [9].

Various subspecialties advocated for different EC techniques based on their training and experience. An ideal EC technique requires minimal steps, is easily taught and replicated, and offers the best chance of success with minimal risk of complications to the patient [10]. Regardless of the technique used, EC involves establishing an airway by incising through the skin and subsequently through the cricothyroid membrane to pass an endotracheal tube into the trachea. While all accepted techniques for performing EC procedure involve these basic steps, various techniques and kits have been developed over the years. In addition, there is little consensus on which technique should be favored as each is associated with variable insertion times, complications, and risks [5, 11]. This study aims to identify the major techniques used to perform EC in a regional trauma center through a retrospective chart review study. Our study also aims to evaluate outcomes associated with the identified techniques. We hypothesized that each specialty has their own preferred method of performing EC based on their clinical training. Furthermore, we hypothesized that there will no difference in mortality or complication rate among different EC techniques.

## Methods

This retrospective study was approved on 7-16-2019 by the Institutional Review Board (IRB) at Arrowhead Regional Medical Center (ARMC) with the IRB approval number 19–33. Informed consent was waived based on the retrospective nature of the study. ARMC is a 456-bed acute care teaching facility located in Colton, California in the United States and is an American College of Surgeons verified level II trauma center in San Bernardino County. ARMC is one of the busiest emergency departments (ED) in the state of California with more than 100,000 visits including more than 3000 adult traumas annually [12]. The study population included trauma patients who presented and underwent an EC at ARMC between 1-1-2009 and 1-1-2019.

All data were collected from the electronic medical record and trauma registry database. The trauma registry database includes all patients who present to the emergency department with a traumatic injury and meet specified International Classification of Diseases (ICD) inclusion criteria. Patients included in this study were identified from the trauma registry database. Data elements extracted from the trauma registry database included: age, gender, pre-procedural Glasgow Coma Scale (GCS), Injury Severity Score (ISS), race/ethnicity, marital status, past medical history, social history, surgical history, initial vital signs, chief complaint, other injuries/symptoms, hospital course, hospital length of stay, disposition, and clinical outcome. Data elements collected from the electronic medical record included: documentation from ED and Trauma history and physical, procedure and progress notes that included indication for cricothyrotomy, technique used, performing physician by subspecialty, location performed, and complications.

Indications for an EC include oral or maxillofacial trauma, cervical spine trauma, profuse oral hemorrhage, copious emesis, or anatomic abnormalities that prevent endotracheal intubation resulting in patients meeting “Cannot Intubate, Cannot Ventilate” criteria [13]. Complications are categorized into early and late complications. Early complications were defined as failure, hypoxia, bleeding, tracheal perforation, fistula formation, scarring, and lacerations to the thyroid, tracheal cartilage, tracheal rings, cricoid, vessels, nerves, esophagus, cartilage, and muscle. Late complications are defined as infection, non-adhesion, subglottic stenosis and voice changes [1]. The decision to perform cricothyrotomy during airway management was made by the attending physician with reference to standardized criteria for the difficult airway. Successful cricothyrotomy was considered to be proper placement of an endotracheal tube or appropriate designated airway devices into the tracheal lumen.

All statistical analyses were conducted using the SAS software for Windows version 9.4 (Cary, North Carolina, USA). Descriptive statistics were presented as means and standard deviations for continuous variables, along with frequencies and proportions for categorical variables. An independent t-test was conducted to assess whether the continuous variables were different between the two techniques. Wilcoxon rank sum tests were utilized to assess the difference between the two techniques in regard to those continuous variables who were not normally distributed. A Chi-square test was then conducted to assess the association of categorical outcomes of the two techniques. If the expected cell counts were less than 5, Fisher’s exact test was conducted to assess the association. All statistical analyses were two-sided. A *p*-value < 0.05 was considered to be statistically significant.

## Results

**Fig. 1:**
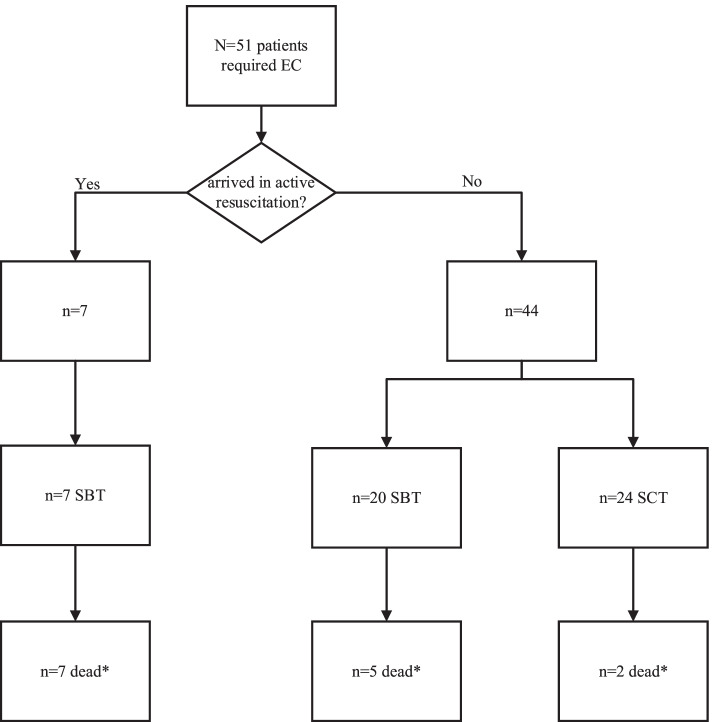
Patient flow chart. *In-depth analysis indicated that none of the mortality was directly related to the EC procedure or complication. EC = emergent cricothyrotomy; SBT = scalpel-bougie-tube; SCT = surgical cricothyrotomy technique

During the ten-year period of this study, ARMC provided care to 29,213 patients who required elective (*n* = 16,645) or emergent intubation (*n* = 12,568) [4]. A total of 51 (0.17%) of these patients required EC and were included in the database. The two major techniques identified were the scalpel-bougie-tube (SBT) technique and the surgical cricothyrotomy technique (SCT). The number of SCT and SBT are roughly equal, with SBT being slightly more frequent (*n* = 27, 52.9%). Figure 1 presented the detailed patients flow chart.

Table 1 presents the demographic summary of these patients. The average patient’s age was 45.2 (SD = 19.93) years, the average GCS was 9.51 (SD = 5.21), and the average ISS was 24.96 (SD = 12.16). A majority of patients were males (76.5%, *n* = 39) and 45.1% (*n* = 23) were Hispanic, which is reflective of the demographics of the region in which ARMC is located, according to the United States Census Bureau [12].

**Table 1:** Comparison of variables between the two techniques

	Overall (***n*** = 51)	Scalpel-bougie-tube (***n*** = 27)	Surgical cricothyrotomy technique (***n*** = 24)	***p***-value
**Age**	45.2 ± 19.93	46.04 ± 19.7	44.25 ± 20.57	0.7528
**Glasgow Coma Scale**	9 (3, 15)	4 (3, 12)	15 (8.5, 15)	0.0036
**Injury severity score**	23 (16, 28)	22 (16, 30)	24.5 (17, 27)	0.896
**Systolic blood pressure**	113.75 ± 49.93	93 ± 54.27	137.08 ± 31.88	0.0009
**Diastolic blood pressure**	66.1 ± 29.76	54.7 ± 35.58	78.92 ± 13.06	0.0023
**Pulse**	83.98 ± 41.65	71.7 ± 49.67	98.39 ± 23.35	0.0173
**Respiratory rate**	17.12 ± 10.23	12.71 ± 12.37	21.52 ± 4.55	0.0051
**Gender**				0.3709
Female	12 (23.5%)	5 (18.5%)	7 (29.2%)	
Male	39 (76.5%)	22 (81.5%)	17 (70.8%)	
**Race**				0.8935
Caucasian	13 (25.5%)	7 (25.9%)	6 (25%)	
African American	12 (23.5%)	7 (25.9%)	5 (20.8%)	
Asian	3 (5.9%)	2 (7.4%)	1 (4.2%)	
Hispanic	23 (45.1%)	11 (40.7%)	12 (50%)	
**Indication for EC**				0.2861
Airway obstruction (pharyngeal mass or angioedema)	12 (23.5%)	4 (14.8%)	8 (33.3%)	
Facial/neck trauma (blunt and penetrating)	23 (45.1%)	14 (51.9%)	9 (37.5%)	
Failed endotracheal intubations	16 (31.4%)	9 (33.3%)	7 (29.2%)	
**Mortality**				0.2172*
Alive	37 (72.6%)	15 (55.6%)	22 (91.7%)	
Patients who arrived at the hospital without vital signs and had resuscitative efforts terminated in the trauma bay within 15 minutes of arrival	7 (13.7%)	7 (25.9%)	0 (0%)	
Dead	7 (13.7%)	5 (18.5%)	2 (8.3%)	
**Complications**				
Early^a^	0	0	0	not applicable
Late^a^	0	0	0	not applicable

*SBT Scalpel-bougie-tube, *SCT* Surgical cricothyrotomy technique, *EC* Emergent cricothyrotomy *The comparison on morality between SBT and SCT excluded 7 patients who arrived at the hospital without vital signs and had resuscitative efforts terminated in the trauma bay within 15 minutes of arrival; ^a^Early complications were defined as failure, hypoxia, bleeding, tracheal perforation, fistula formation, scarring, and lacerations to the thyroid, tracheal cartilage, tracheal rings, cricoid, vessels, nerves, esophagus, cartilage, and muscle. Late complications are defined as infection, non-adhesion, subglottic stenosis and voice changes

Table 1 also presents the comparison of variables between the two techniques. Overall, there was no statistically significant difference between the two techniques with regards to demographic variables, including age (*p* = 0.7528), ISS (*p* = 0.896), gender (*p* = 0.3709), and race (*p* = 0.8935). However, the SCT group had a statistically higher than the SBT group in GCS (*p* = 0.0036), systolic blood pressure (*p* = 0.0009), diastolic blood pressure (*p* = 0.0023), pulse (*p* = 0.0173), and respiratory rate (*p* = 0.0051). There was no statistically significant difference in mortality between these two groups (*p* = 0.2172). There were no complications documented with any of the cricothyrotomies identified.

## Discussion

**Fig. 2:**
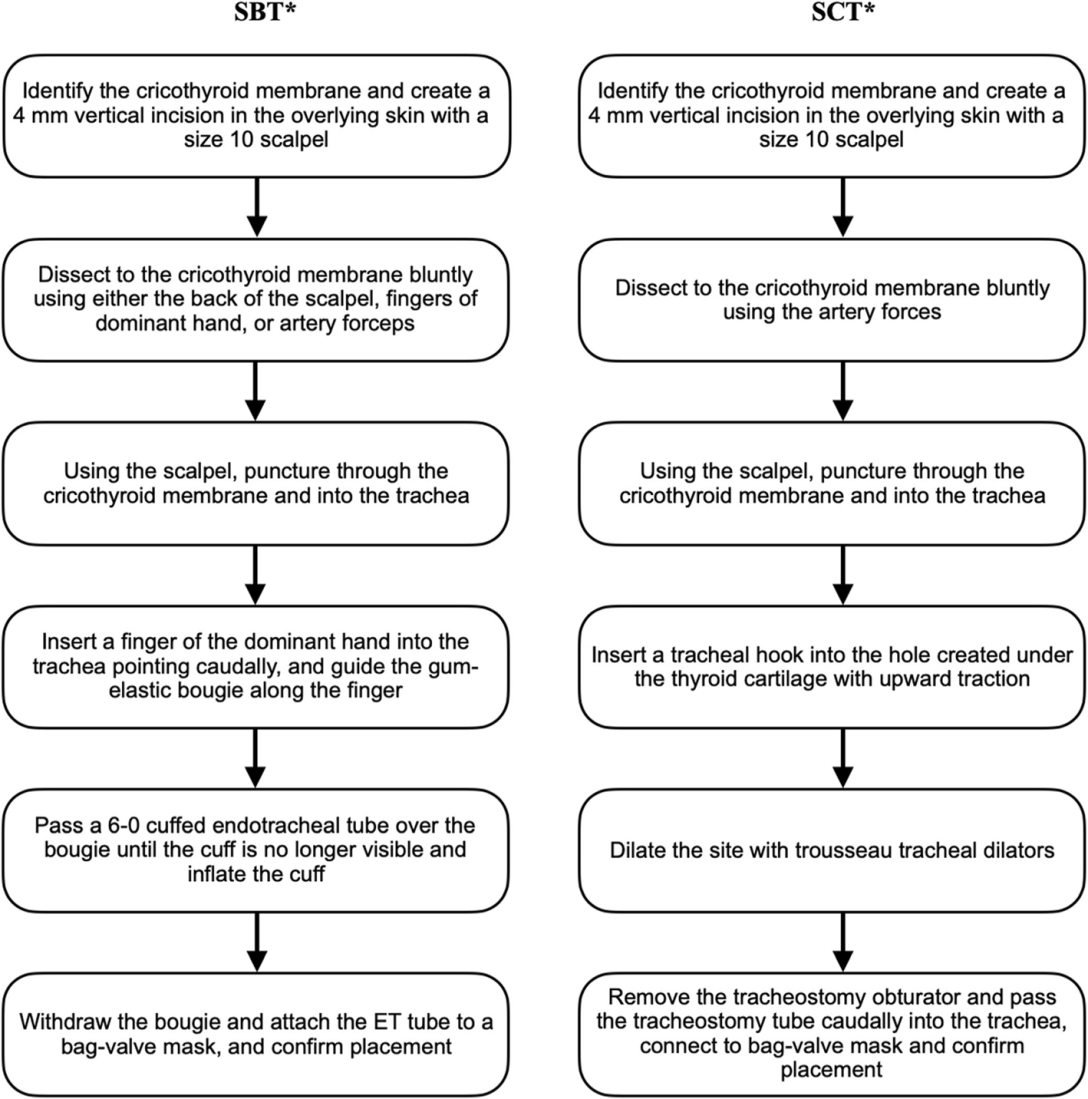
Steps to perform the two major emergency cricothyrotomy procedures. *SBT = scalpel-bougie-tube technique; SCT = surgical cricothyrotomy technique

An EC is a surgical technique reserved for failed airways in adults. It is a rare and crucial procedure in the emergency airway management algorithm of a “cannot intubate, cannot oxygenate” scenario [1]. Numerous techniques have been described, but reaching a consensus on the best technique is difficult due to the scarcity of data [10]. Our review revealed two primary techniques performed within ARMC, namely the SBT and SCT techniques. Both techniques are discussed in detail in Fig. 2. The SBT technique requires a scalpel and a bougie while the SCT method requires a hook and dilator. The availability of the medical equipment in a particular setting may determine which technique was performed.

There are approximately five ECs performed per year at ARMC. Due to the critical nature and rarity of this procedure, data have been limited to studies involving cadaver and animal models. Additionally, anatomical variation and inherent differences between these models introduce further bias and confounding factors. Therefore, a proper comparison between these two techniques to determine superiority is difficult [10]. Our study found no significant difference between the two techniques with regards to patient outcomes, as both accomplished the goal of securing an emergency airway without complications. This is consistent with other researchers, who found all utilized techniques to be successful in establishing an emergency airway [14]. There were no early or late complications noted during the procedure or hospital stay in our cohort. Similar findings were reported by other researchers [15, 16]. .It is worth noting that McGill and colleagues reported that EC related complications rate up to 31.6% in a prehospital setting [17]. The difference in the complication rate between prehospital and hospital setting may be attributed to the higher experience of physicians and controlled environment.

The rate of each EC technique in our study was nearly equal, with 53% using SBT and 47% using SCT. SBT was performed exclusively by ED physicians while SCT was performed exclusively by surgeons. This aligns with previous literature reporting SBT being taught in emergency medicine training programs, while SCT is the preferred method promoted by the American College of Surgeons [1, 10, 18–20]. Hamaekers et al. noted no consensus on the best technique or device for the emergency percutaneous airway [10]. It has been postulated that ED physicians prefer SBT as it requires fewer steps to secure an emergency airway. By contrast, surgeons may prefer SCT due to the nature of their training which allows more time with surgeries and more experience with the involved tools [13]. Other factors that may impact the decision are patients’ anatomy, available medical equipment, complications from previous attempts, and a prolonged intubation course.

No deaths were directly due to EC in our experience. Deaths occurred either following termination of continued prehospital cardiac resuscitation, all of which ceased within 15 minutes of arrival to ARMC (7/51, or 13.7%), or occurred during hospitalization and were not clearly related to EC procedure (7/51, or 13.7%). The impact of the EC technique on mortality is generally difficult to assess as the majority of these patients are critically ill. Kwon and colleagues similarly found that the majority of patients who underwent EC expired as a result of their primary illness, regardless of the technique used [5]. The two techniques utilized at ARMC can be considered equivalent with regards to initial morbidity and mortality in our experience.

Since emergency airway management is part of the initial resuscitation process, time to completion for EC is usually poorly documented and has so far been predominantly recorded in studies focused on cadavers, animals, and mannequin models [18, 21]. In this study we noted a similar pattern of poorly documented procedural timelines. The SBT method has been shown to be faster and less complicated for the inexperienced provider in an animal study, though this may not be an adequate comparison in the context of real-world patient care [18]. Herring and colleagues compared average EC times on human mannequins using SCT versus SBT techniques. They noted an average procedure time of 86.6 seconds for SBT and 130 seconds for SCT [22]. Similar findings were also reported by prior studies that noted SBT may be faster than SCT in practice. However, the available data are very limited [13, 21]. By contrast, using SBT may also be more technically difficult in obese patients with large amounts of adipose tissue, possibly making SCT a more sensible choice in such cases [23]. Crosby et al. suggests that the most important predictor of success for either technique is familiarity and technical mastery, particularly given the rarity of the procedure and concern for skill decay [24]. This is also supported by our data as ED physicians preferred SBT, and surgeons preferred SCT.

The Difficult Airway Society guidelines recommend SBT as the first-line technique for EC, noting it to be fast and streamlined. This is ideal in critical situations, when the stress can diminish cognitive processing and motor skills [2]. In an acute setting such as the ED, SBT can be reliably used to increase the odds of patient survival. In addition, using a cuffed endotracheal tube offers several advantages including the ability to ventilate using standard low-pressure airway equipment, and confirming correct placement using capnography [23]. It is also widely believed that the cuffed endotracheal tube may also protect against aspiration [25].

A significant limitation to this study is that data collection was primarily based on chart review of patients from the electronic medical records. Therefore, any patients who may have had EC performed, but were not listed with an ICD-9 or 10 code reflecting that procedure, may have been overlooked in our review. Secondly, another notable issue is that physicians and/or their scribes had varying amounts of documentation regarding information around the EC procedure itself, including the number of intubations attempted, details of the technique used, and notably the total time of the procedure. Lastly, because of the time constraints and acuity of the condition, other data points might not have been documented such as minor complications related to the procedure.

## Conclusion

We reviewed practices in a busy Southern California trauma center and found two preferred techniques for performing EC, the SBT and the SCT. In general, ED physicians utilized SBT more frequently, while surgeons utilized SCT more often. This may be due to variations in education and familiarity. We found no difference in the rate of success or complications between the two techniques, and no deaths were directly due to EC. Practitioners may choose a preferred technique and be well versed in that technique given the rarity of the procedure.

## Data Availability

The datasets generated during and/or analyzed during the current study are available from the corresponding author on reasonable request.

## Abbreviations

ARMC: Arrowhead Regional Medical Center
EC: Emergent cricothyrotomy
ED: Emergency department
GCS: Glasgow Coma Scale
ICD: International Classification of Diseases
ISS: Injury severity score
SBT: Scalpel-bougie-tube
SCT: Surgical cricothyrotomy technique
